# Standalone sauna vs exercise followed by sauna on cardiovascular function in non‐naïve sauna users: A comparison of acute effects

**DOI:** 10.1002/hsr2.393

**Published:** 2021-10-01

**Authors:** Earric Lee, Joel Kostensalo, Peter Willeit, Setor K. Kunutsor, Tanjaniina Laukkanen, Francesco Zaccardi, Hassan Khan, Jari A. Laukkanen

**Affiliations:** ^1^ Faculty of Sport and Health Sciences University of Jyväskylä Jyväskylä Finland; ^2^ Faculty of Mathematics and Science University of Jyväskylä Jyväskylä Finland; ^3^ Department of Neurology Medical University of Innsbruck Innsbruck Austria; ^4^ Department of Public Health and Primary Care University of Cambridge Cambridge UK; ^5^ National Institute for Health Research Bristol Biomedical Research Centre University Hospitals Bristol and Weston NHS Foundation Trust and the University of Bristol Bristol UK; ^6^ Translational Health Sciences, Bristol Medical School University of Bristol, Learning & Research Building (Level 1), Southmead Hospital Bristol UK; ^7^ Institute of Public Health and Clinical Nutrition University of Eastern Finland Kuopio Finland; ^8^ Diabetes Research Centre Leicester General Hospital Leicester UK; ^9^ Division of Cardiology, Department of Medicine Emory University Atlanta Georgia USA; ^10^ Department of Internal Medicine Central Finland Health Care District Jyväskylä Finland; ^11^ Institute of Clinical Medicine University of Eastern Finland Kuopio Finland

**Keywords:** aerobic exercise, arterial compliance, hypertension, lifestyle modification, non‐pharmocological therapy

## Abstract

**Background and aims:**

Sauna bathing and aerobic exercise have each been shown to affect cardiovascular function. However, direct comparisons between standalone sauna bathing and a combination of exercise and sauna on vascular indices remain limited. Therefore, we conducted a cross‐over study using matched durations to explore the hemodynamic changes of sauna exposure when compared to a combination of aerobic exercise and sauna exposure.

**Methods:**

Participants (N = 72) with at least one cardiovascular risk factor underwent, on two separate occasions: (a) a 30‐minute sauna at 75°C (SAUNA) and (b) the combination of a 15‐minute cycling exercise at 75% maximum heart rate followed by 15‐minute sauna exposure (EX+SAUNA). Relative changes to arterial stiffness (PWV), augmentation index (Alx), brachial systolic and diastolic blood pressure (SBP and DBP), central SBP (cSBP), mean arterial pressure (MAP), and heart rate (HR) were compared PRE‐POST and pre‐ to 30‐minutes post‐intervention (PRE‐POST30).

**Results:**

Baseline SBP and DBP were 143 (SD 18) mmHg and 86 (SD 10) mmHg, respectively. From PRE‐POST, SAUNA had lower DBP (mean difference [95% CI] 2.5 [1.0, 4.1], *P* = .002) and MAP (2.5 [0.6, 4.3], *P* = .01). However, EX+SAUNA had lower SBP (−2.7 [−4.8, −0.5], *P* = .02), DBP (−1.8 [−3.3, −0.4], *P* = .01), and MAP (−2.0 [−3.5, −0.5], *P* = .009) PRE‐POST30. There were no statistically significant differences between SAUNA and EX+SAUNA for other measured parameters.

**Conclusion:**

This study demonstrated that when matched for duration, EX+SAUNA and SAUNA elicit comparable acute hemodynamic alterations in middle‐aged participants with cardiovascular risk factors. The sauna is a suitable option for acute blood pressure reductions in those who are unable to perform aerobic exercise, and may be a viable lifestyle treatment option to improve blood pressure control.

## 1 INTRODUCTION

Sauna bathing has been associated with a lower risk for cardiovascular disease (CVD) outcomes,^1^ improved vascular endothelial and cardiac function,^2^ lower blood pressure,^3^ and positive alterations in several hemodynamic markers.^4^ Sauna bathing exerts a strain on the cardiovascular system, to maintain blood pressure and sufficient blood flow to other organs and muscles.^5^ A recent meta‐analysis^6^ found sauna use to play a positive role in improving cardiovascular function and functional capacity as well.

Likewise, aerobic exercise has been well documented to provide cardiovascular^7^ and disease prevention^8^ benefits, especially for older adults^9^ and populations with adverse levels of risk.^10^ Furthermore, aerobic exercise has also been shown to acutely reduce arterial stiffness,^11^ although a recent study^12^ suggests that improvements in arterial stiffness are only seen when an impairment is present. Arterial stiffness is a useful marker in the prediction of cardiovascular events and has been shown to be capable of predicting cardiovascular mortality independent of other traditional markers.^13^ Nevertheless, several studies have noted the comparable physiological effects of sauna bathing and aerobic exercise.^14^, ^15^

Previous research investigated sauna bathing as a post‐exercise intervention with promising results,^16^, ^17^ but the mechanisms of action for both modalities were not directly comparable due to methodological differences. Moreover, although some studies have investigated the conjunctive use of exercise and sauna in athletes,^18^, ^19^ this information remains somewhat limited in non‐athlete populations. However, Rosenberg and colleagues^20^ did speculate about the usefulness of adjunctive exercise and heat therapy, which was partially shown by our group,^21^ where a combination of 15 minutes of aerobic exercise followed by 15 minutes of sauna bathing showed several notable improvements to hemodynamic function.

Long‐term sauna therapy has been shown to effectively improve exercise tolerance,^22^ while regular exercise and sauna exposure were able to improve cardiac function and autonomic nervous system activity,^23^ albeit in populations with heart‐related issues. Therefore, there is reason to believe that the combination of exercise and heat therapy in the form of sauna bathing may indeed complement each other. Both current and previous literature^24^, ^25^ are in support of this postulation, demonstrating heat augmented physiological responses. As such, a comparison of the effects between these two interventions using an equal exposure time of 30 minutes will help to further elucidate any therapeutic potential that may exist, given the benefits seen from 30 minutes of acute^26^ and regular aerobic exercise.^27^

To the best of our knowledge, changes to arterial stiffness and hemodynamics between sauna bathing, and a combination of aerobic exercise followed by sauna bathing have yet to be investigated. Our group has previously explored the effects of acute sauna exposure,^4^ and thus aim to extend our findings and compare the effects of sauna exposure alone against a shortened duration of exercise plus sauna exposure. This may be of considerable benefit for a broader population, including those who have lower exercise capacities.^28^ Therefore, the purpose of the study was to explore the hemodynamic changes of a single session of sauna exposure compared to the combination of aerobic exercise and sauna exposure of matched duration, in a population with at least one cardiovascular risk factor.

## 2 METHODS

### 2.1 Participants

Participants (N = 72; females = 33, males = 39) were recruited from the city of Jyväskylä, Central Finland, through the local out‐of‐hospital health care center. To be eligible for inclusion, participants had to be free of a prior diagnosis of CVD and exhibited at least one of the following cardiovascular risk factors: a history of smoking, hyperlipidemia, hypertension, clinically diagnosed diabetes, obesity, or family history of coronary heart disease (CHD). Hypercholesterolemia was defined as the use of cholesterol drugs or serum low‐density lipoprotein cholesterol over 3.5 mmol/L. Hypertension was defined as having a systolic reading of greater than 140 mm Hg, and/or a diastolic reading of greater than 80 mm Hg on two or more separate resting measurements. Obesity was defined as body mass index >30 kg/m^2^. Family history of CHD was considered positive if father (<55 years) or mother (<65 years) had premature CHD. Menstrual status of female participants was not taken into account during recruitment; but none of them were menstruating at the time of the administration of interventions. Among the female participants, 9 were premenopausal, while 24 were postmenopausal. All participants provided written informed consent and were informed about the research purposes and measurement procedures, before being screened by a cardiac specialist. The research protocol and study design were approved by the institutional review board of the Central Finland Hospital District ethical committee, Jyväskylä, Finland (Dnro 5U/2016).

### 2.2 Experimental design

**FIGURE 1:**
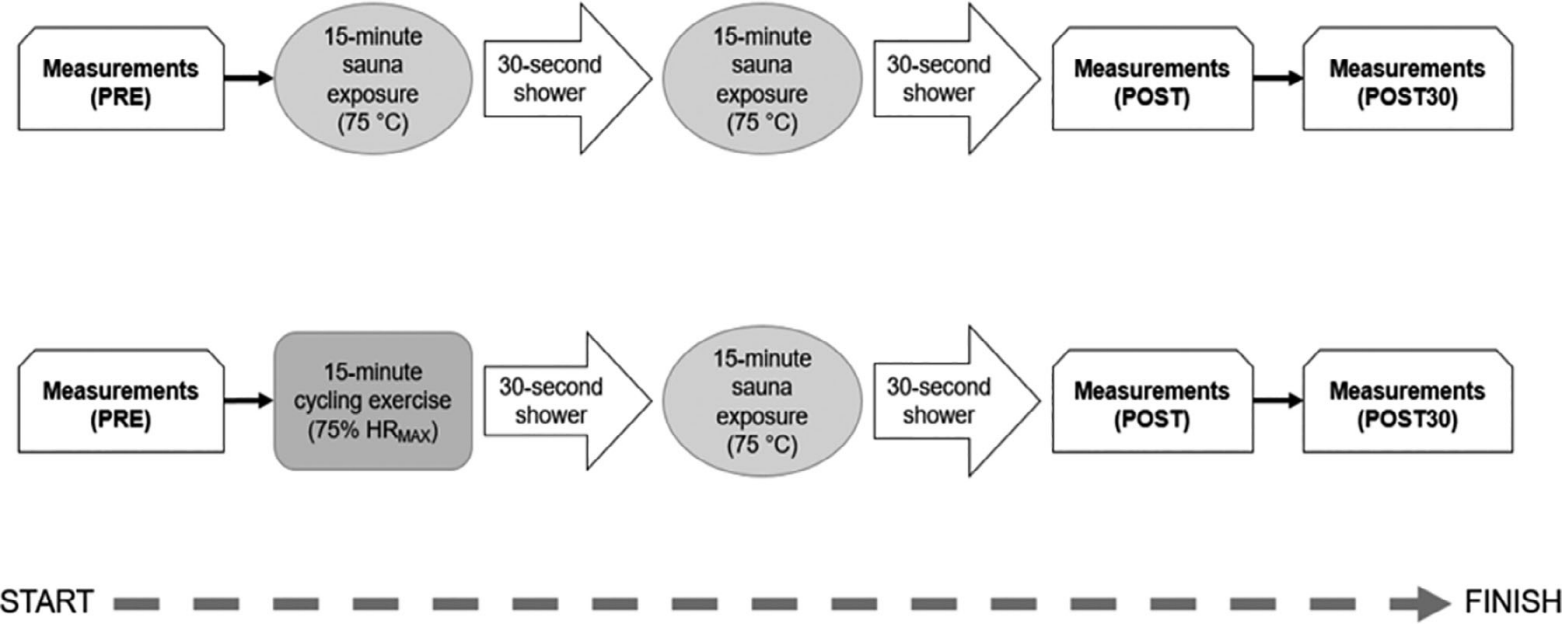
Experimental design flow and order

Figure 1 is an overview of the study design. In this balanced nonrandomized crossover trial, all study participants underwent two interventions, each on a separate occasion (>72 hours apart) between 1000 and 1600. A standalone 30‐minute sauna at 75°C (SAUNA), and 15‐minutes of cycling on a stationary bike at 75% of individual maximum heart rate, followed by 15‐minutes of sauna exposure (EX+SAUNA). A cycling exercise test was conducted on a separate day prior to the experiment to ascertain individual maximal exercise heart rates, which was then used to calculate individual 75% maximum. The exercise test was conducted on an electromagnetically braked cycle ergometer (Monark Exercise AB, Sweden) utilizing a graded exercise test protocol with continuous electrocardiogram recordings (CardioSoft software V.1.84, GE Healthcare, Freiburg, Germany). The symptom‐limited exercise test was started with 5‐minute warm‐up without workload for each participant and continued with 20‐W increments applied every 1 minute until volitional exhaustion. All exercise tests were conducted under the supervision of a qualified nurse and physician and the same bike was used for the experiment.

After the first 15‐minute period of SAUNA, the participants left the sauna room to have a quick shower (<30 seconds) before going back for the second 15‐minute period. Participants were in a seated position during all sauna sessions, and the same sauna room (75°C) was used for all participants. Cycling exercise was conducted within 10 m of the sauna room to minimize transit time during EX+SAUNA. Participants were instructed to keep the cadence between 65 and 70 rpm for the entire 15 minutes. The cycling load in watts was monitored and adjusted throughout the duration of the exercise to ensure that the heart rate for each participant was kept at 75% of their individual maximum exercise heart rate, pre‐calculated using data obtained from the exercise test.

The temperature and humidity of the exercise room were 21°C and 25%, respectively. Participants were instructed to abstain from eating 2 hours, caffeine 12 hours, alcohol 24 hours, and exercise and/or sauna 48 hours prior to the measurements. Food intake was not standardized. Fluid was consumed ad libitum. A physician was in attendance at all times and participants were allowed to leave the sauna or stop the experiment at any time if they felt uncomfortable, but all participants underwent the two interventions successfully.

### 2.3 Assessment of outcome measures

The measurements of arterial stiffness during this experiment adhered closely to published guidelines.^29^ Supine brachial SBP and DBP were obtained using Microlife BP A200 (Microlife Corp., Taipei, Taiwan). Two sequential readings were taken and the mean values were used. All measurements were done on the right side of the body with the participant in the supine position. Transit distances were assessed by body surface measurements using a tape measure. There was a total of three transit distances, carotid artery site to suprasternal notch; carotid artery to femoral artery; and suprasternal notch to femoral artery. Carotid to femoral measurement was adjusted to 80% (common carotid artery − common femoral artery × 0.8) for the calculation of PWV as recommended.^29^

Brachial blood pressures and PWV as a measure of arterial stiffness were taken in their respective order at three different time points; before (PRE), immediately after (POST), and after a 30‐minute recovery (POST30). All measurements were taken by a single trained operator of the tonometer to minimize ascertainment biases and to ensure consistency and reliability (ICC 2.1:0.75 with 95% confidence interval [CI] = 0.63‐0.83, SEM = 0.4). ICC estimates and their 95% CIs were calculated using two repeated measurements taken 10 days apart (prior to the experiment) from 72 participants via the statistical software R,^30^ based on a mean‐rating (*k* = 2), absolute‐agreement, two‐way mixed‐effects model. High‐quality recordings (defined as an in‐device quality index of more than 90% from an average of at least 10 cardiac cycles) were collected using the PulsePen device (DiaTecne s.r.l., Milan, Italy; www.pulsepen.com) using methods that have been documented in previous studies.^4^, ^21^ PWV, augmentation index (AIx), central systolic blood pressure (cSBP), and mean arterial pressure (MAP) were subsequently estimated via the software.

Participants were permitted to take a quick shower (<30 seconds) before POST measurements were taken. Water temperature of the shower was not controlled and participants could freely select their desired temperature. Thereafter, they were instructed to rest in a designated waiting lounge (21°C, humidity 25%) in a seated position for a duration of 30 minutes before the final measurement (POST30) was taken. Participants were kept in a supine position for 7 minutes prior to the measurements.

### 2.4 Statistical analyses

The individual treatment effects, that is, the differences between pre‐ and post‐intervention and follow‐up measurements (PRE, POST, POST30) do not a priori follow a normal distribution. This was confirmed by 30% of the Shapiro‐Wilk normality tests for PRE, POST, and POST30 effects giving *P*‐values below .05. Graphical investigations revealed no remarkable differences in the distribution of responses related to age or gender, so controlling for these variables still leaves us with non‐Gaussian distributions. As such, the assumptions of the basic parametric approaches, such as Student's *t* test and ANOVA were not satisfied. Therefore, a nonparametric approach was selected. Statistical inference in this work is based on the Neyman‐Rubin causal model.^31^, ^32^, ^33^ For the variable *Y,* the causal effect of treatment *T* with respect to control *C* for the individual *u* is defined as

$$Y_{T}\left(u\right)-Y_{C}\left(u\right).$$

The effects of the two different interventions (S and ES) for the same individual cannot be simultaneously measured. This is known as the *fundamental problem of causal inference* in the Neyman‐Rubin model. We solve the fundamental problem using a combination of scientific and statistical solutions^31^ by assuming temporal stability (the responses of the individuals would be the same if the treatments would have been done in the opposite order) and then calculating the average effect of EX+SAUNA relative to SAUNA. The temporal stability assumption was investigated by performing a sign test on the PRE measurements. No statistically significant differences were found in the PRE measurements between EX+SAUNA and SAUNA.

Stable unit treatment value is also assumed, that is, the outcome of one individual does not depend on the interventions done on the other individuals, which is contextually natural. The CI and *P*‐value were estimated using nonparametric bootstrap^34^ with 100 000 simulations for each response. A two‐tailed alternative hypothesis was assumed as we did not have any reason to be certain about the *direction of the difference* for any of the response variables. In the nonparametric bootstrap method, we resample the empirical distribution of responses by taking random samples equal in size to the original sample (N = 72) with replacement. The resulting distribution asymptotically approaches the true sampling distribution. As N = 72 the bootstrap samples are drawn from a representative approximation for the population sampling distribution. The analysis consisted of multiple tests for differences between interventions, so we use adjusted *P*‐values for inference, where the adjusting has been done using the Benjamini‐Hochberg method.^35^ Unadjusted *P*‐values are also reported for the purpose of completeness and meta‐analyses.

The reliability of the statistical tests run depends on the validity of the assumption that the participants can be analyzed as a single group. However, it is possible that the responses might be dependent on for example, the age or biological sex of the participant, although this has largely been controlled for with the crossover study design and the inclusion/exclusion criteria. Nonetheless, we ran the analyses in several subgroups to ascertain whether the assumption of homogeneity holds. The analysis was for age, biological sex, initial SBP over 140 or DBP over 80, and menopausal status. Similar responses were observed in each subgroup. Based on these assessments we concluded that controlling for these covariates would not make any noticeable difference in this data set.

The full‐sample comparisons were pre‐specified, with a priori determined significance level of *P* < .05 to be used for the adjusted *P*‐values along with a two‐tailed alternative hypothesis. The subgroup analyses and normality tests were exploratory in nature, and were aimed at determining whether parametric tests or models with covariates could be adopted in order to increase the statistical power. Two‐tailed alternative hypotheses were assumed here as well. The calculations were implemented with the statistical software R version 3.6.3^30^ with the plots done using the ggplot2 package.^36^ Continuous data are presented as means ± SD and categorical data as frequencies (percentage of the whole). To ensure that the statistical analyses were nonbiased, variables were coded and analyzed by an independent statistician who was completely blind to the experiment. In addition, the statistician carrying out the analyses was completely uninvolved in the participant recruitment and data collection processes.

## 3 RESULTS

### 3.1 Characteristics of participants

The characteristics of the participants are presented in Table 1. Mean baseline SBP and DBP were 143 and 86 mm Hg, respectively. The three cardiovascular risk factors most commonly present were hyperlipidemia (71%), family history of CHD (43%), and obesity (31%).

**TABLE 1:** Participant characteristics

Normally distributed parameters	Mean ± SD (N = 72)
Age (years)	54 ± 9
Body mass (kg)	83.2 ± 15.0
Body mass index (kg/m^2^)	27.8 ± 4.3

Non‐normally distributed parameters	Median; IQR
Resting SBP (mm Hg)	135; 21
Resting DBP (mm Hg)	83; 14
Resting heart rate (b/min)	66; 11

Sauna habits	Number (%)
Less than once a week	15 (21)
Once a week	15 (21)
Two to three times per week	25 (35)
Four or more times per week	17 (23)

Aerobic exercise frequency	Number (%)
Less than once a week	23 (32)
Once a week	15 (21)
Two to three times per week	19 (26)
Four or more times per week	15 (21)

Categorical parameters	Number (%)
Smoker (history of smoking)	12 (17)
Hyperlipidemia	51 (71)
Hypertension	15 (21)
Diabetes^a^	3 (4)
Obesity (body mass index > 30)	22 (31)
Family history of coronary heart disease	31 (43)

Abbreviations: DBP, brachial diastolic blood pressure; SBP, brachial systolic blood pressure.

^a^ For diabetes, two were type 2 diabetes and one was type 1 diabetes.

### 3.2 Changes in outcome measures from PRE to POST

**FIGURE 2:**
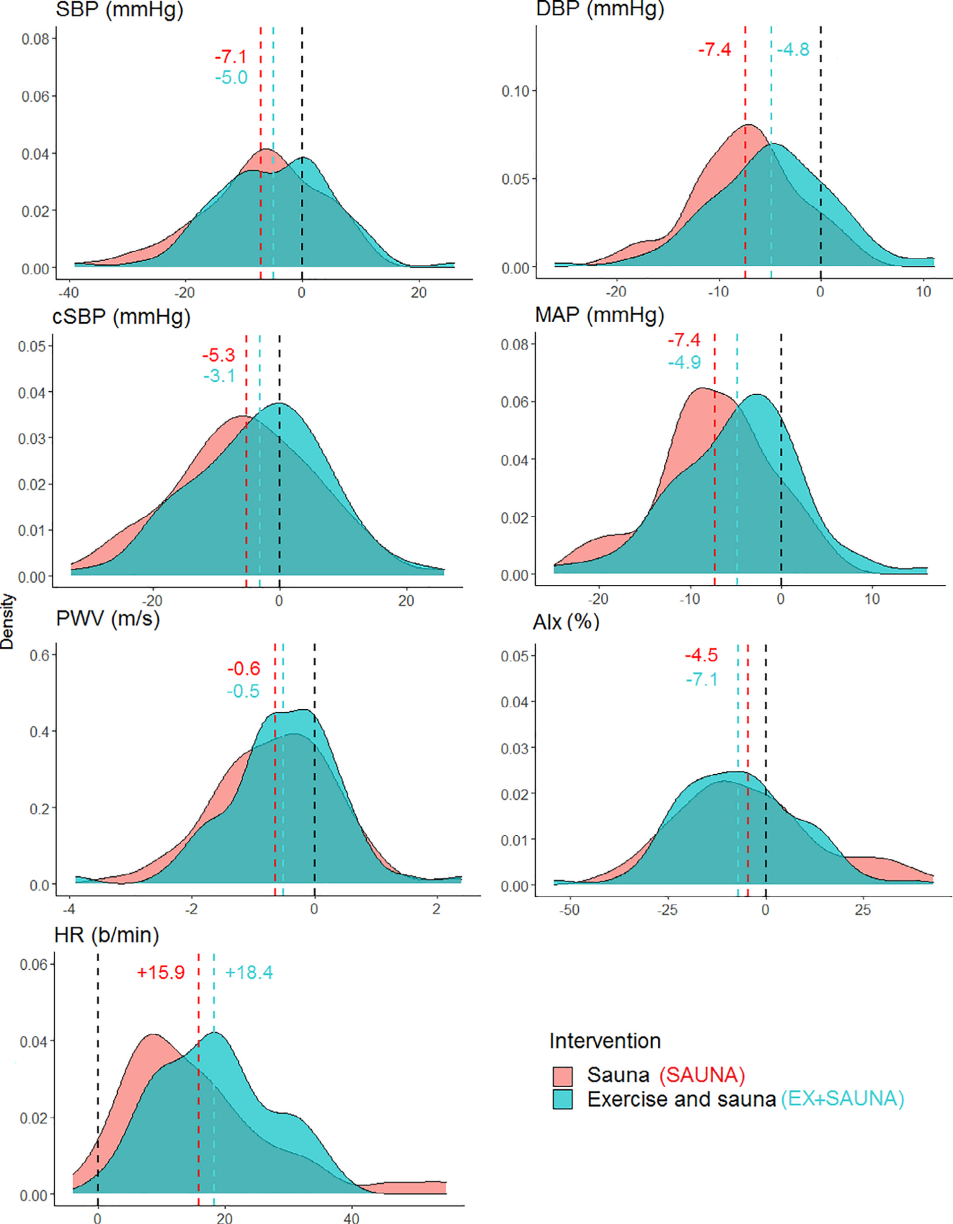
The distribution of individual responses to the two interventions as measured between PRE and POST

PRE‐POST changes in outcome measures within and between interventions, with their corresponding average Neyman‐Rubin causal effect (difference between EX+SAUNA and SAUNA) are shown in Table 2. DBP and MAP were significantly lower for SAUNA. However, there was no statistically significant PRE‐POST difference between EX+SAUNA and SAUNA for other outcome measures. The distribution of individual responses to the two interventions between PRE and POST is displayed in Figure 2.

**TABLE 2:** PRE‐POST comparison

Parameters	EX+SAUNA		SAUNA		Average CE	95% CI	Raw *P*‐value	Adjusted *P*‐value
	Mean	95% CI	Mean	95% CI				
PWV (m/s)	−0.5	(−0.7, −0.3)	−0.6	(−0.9, −0.4)	0.1	(−0.2, 0.4)	.436	.46
AIx (%)	−7.1	(−10.6, −3.6)	−4.5	(−8.6, −0.2)	−2.6	(−7.7, 2.6)	.323	.38
SBP (mm Hg)	−5.0	(−7.4, −2.6)	−7.1	(−9.4, −4.8)	2.1	(−0.4, 4.7)	.103	.16
DBP (mm Hg)	−4.9	(−6.3, −3.4)	−7.4	(−8.6, −6.2)	2.5	(1.0, 4.1)	.002	.02
cSBP (mm Hg)	−3.1	(−5.5, −0.8)	−5.3	(−7.9, −2.7)	2.2	(−0.6, 4.9)	.120	.16
MAP (mm Hg)	−4.9	(−6.5, −3.3)	−7.4	(−8.8, −5.9)	2.5	(0.6, 4.3)	.010	.05
HR (b/min)	18.4	(16.3, 20.4)	15.9	(13.0, 18.7)	2.5	(−0.5, 5.5)	.100	.16

*Note*: Average causal effect (CE) is the difference between EX+SAUNA and SAUNA.

Abbreviations: AIx, augmentation index; CE, causal effect; CI, confidence interval; cSBP, central systolic blood pressure; DBP, brachial diastolic blood pressure; HR, heart rate; MAP, mean arterial pressure; PWV, pulse wave velocity; SBP, brachial systolic blood pressure.

### 3.3 Changes in outcome measures from PRE to POST30

**FIGURE 3:**
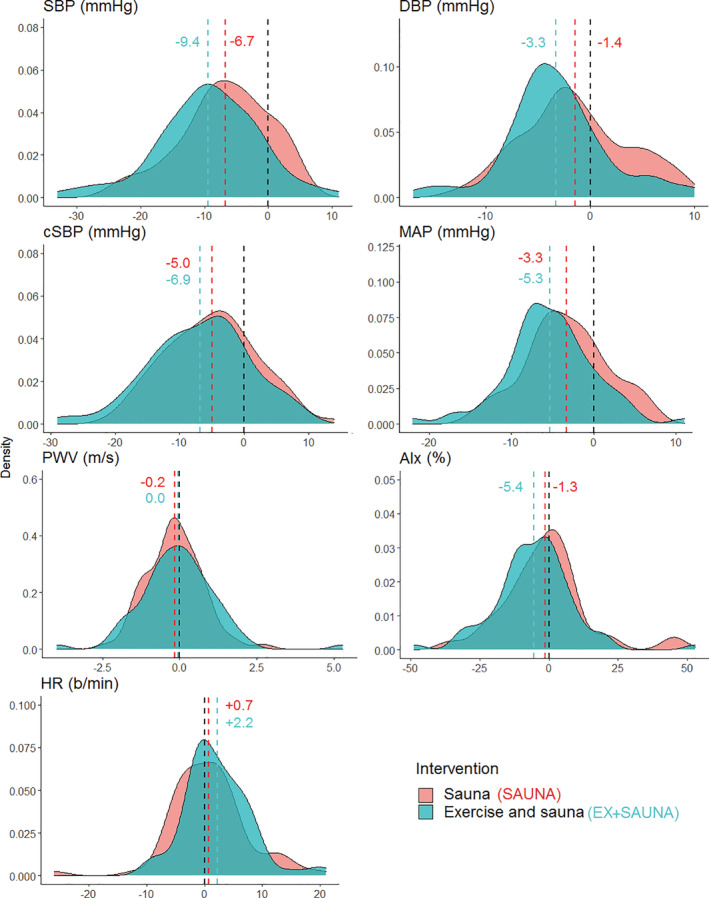
The distribution of individual responses to the two interventions as measured between PRE and POST30

PRE‐POST30 changes in outcome measures and their average causal effect are shown in Table 3. SBP, DBP, and MAP were lower in EX+SAUNA. The distribution of individual responses to the two interventions as measured between PRE and POST30 is displayed in Figure 3.

**TABLE 3:** PRE‐POST30 comparison

Parameters	EX+SAUNA		SAUNA		Average CE	95% CI	Raw *P*‐value	Adjusted *P*‐value
	Mean	95% CI	Mean	95% CI				
PWV (m/s)	−0.0	(−0.3, 0.3)	−0.2	(−0.4, 0.0)	0.1	(−0.2, 0.5)	.548	.55
AIx (%)	−5.4	(−8.6, −2.0)	−1.3	(−4.8, 2.2)	−4.0	(−7.6, −0.5)	.023	.05^a^
SBP (mm Hg)	−9.4	(−11.3, −7.6)	−6.7	(−8.4, −5.2)	−2.7	(−4.9, −0.5)	.018	.05
DBP (mm Hg)	−3.3	(−4.4, −2.1)	−1.4	(−2.6, −0.3)	−1.8	(−3.3, −0.4)	.013	.04
cSBP (mm Hg)	−6.9	(−8.7, −5.1)	−5.0	(−6.6, −3.4)	−1.9	(−4.2, 0.4)	.109	.16
MAP (mm Hg)	−5.3	(−6.6, −4.1)	−3.3	(−4.4, −2.1)	−2.0	(−3.5, −0.5)	.009	.04
HR (b/min)	2.2	(0.9, 3.5)	0.7	(−0.8, 2.3)	1.5	(−0.4, 3.4)	.128	.16

*Note*: Average causal effect (CE) is the difference between EX+SAUNA and SAUNA.

Abbreviations: AIx, augmentation index; CE, causal effect; CI, confidence interval; cSBP, central systolic blood pressure; DBP, brachial diastolic blood pressure; HR, heart rate; MAP, mean arterial pressure; PWV, pulse wave velocity; SBP, brachial systolic blood pressure.

^a^ The actual adjusted value did not reach statistical significance at .054.

## 4 DISCUSSION

In this study, we compared the effects of sauna bathing alone against a short 15‐minute bout of aerobic exercise followed immediately by the sauna, with a matched total duration of 30 minutes for both interventions. Individually, both interventions showed positive alterations PRE‐POST as well as PRE‐POST30. More specifically, SAUNA had significantly lower DBP and MAP between PRE‐POST. However, SBP, DBP, and MAP were lower for EX+SAUNA PRE‐POST30.

One of the first studies comparing between EX+SAUNA and SAUNA in a similar population group demonstrated that EX+SAUNA reduced SBP whereas no change was seen in DBP.^16^ Additionally, SAUNA had no significant effect on blood pressures. The authors speculated that this might have been due to the insufficient sauna stimulus, which was likely to have been the case; relative to the outcomes seen from our present investigation. We used matched durations for both EX+SAUNA and SAUNA which meant that our sauna exposure time was nearly twice as long compared to the study by Gayda et al.^16^ Consequently, this led to a relatively more even comparison as can be seen from our results.

Our results showed that SAUNA induced greater changes PRE‐POST compared to EX+SAUNA in both DBP and MAP. Moreover, all pressure‐related markers had a clinically relevant reduction of 5 mm Hg or more. This was rather unforeseen, as lower values for EX+SAUNA would have been a more likely outcome due to the prevailing literature on post‐exercise hypotension. Nevertheless, the comparability of PRE‐POST hemodynamic responses is in support of the findings from a more recent study, which postulated that the cardiovascular stress from exposure to sauna might be similar to that of moderate aerobic exercise.^15^ Indeed, the decreases in blood pressures seen in EX+SAUNA from this study were comparable to aerobic exercise of longer durations,^37^ despite comprising of only 15 minutes of moderate intensity cycling.

Passive heat exposure has been suggested to improve endothelial function and nitric oxide bioavailability through enhanced dilation of the arterial tree.^38^ This is comparable to how exercise increases dilation of arteries supplying skeletal muscle, which has been documented to lead to reduced wave reflection.^39^ The efficacy of heat stress from the sauna was inadvertently shown in a more recent study,^40^ which sought to utilize the sauna as a post‐training recovery tool. Instead of providing a recuperative effect, performance measures were decreased. The authors postulate that this might have been due to the physiological stress from sauna exposure acting like a further stimulus of exhaustion. This is consistent with earlier studies^17^, ^19^ that have identified sauna exposure as an additional source of training stimuli for athletic populations. Indeed, a more recent study^41^ even considered post‐training sauna use as an effective ergogenic aid.

Our findings are also in agreement with results seen by other researchers. Thomas and coworkers^42^ showed that 30 minutes of heat therapy induced a shear stress response that led to greater decreases in blood pressure than 30 minutes of aerobic exercise, while our current study found lower pressures PRE‐POST from SAUNA compared to EX+SAUNA. One possible explanation for that may be the increase in skin blood flow during heat exposure, which led to decreased peripheral resistance and consequently lower blood pressures. However, peripheral resistance was not one of our outcome measures and the lack of significant concomitant decreases in PWV as a measure of arterial stiffness partially refutes that supposition. Furthermore, both EX+SAUNA and SAUNA had similar PWV nonsignificant responses throughout the experiment (Tables 2 and 3), despite reductions in majority of the hemodynamic parameters.

The lack of significant changes in PWV for EX+SAUNA is in line with a recent review,^43^ which reported no change in PWV after acute aerobic exercise of varying durations. However, this was somewhat of an unexpected finding for SAUNA, as our group had previously shown reductions in PWV.^4^ Nonetheless, these results suggest that arterial wall property change was unlikely to have been the cause of the hypotension in this study, and that other mechanisms^44^ may have been responsible. Although SAUNA induced greater changes PRE‐POST compared to EX+SAUNA, PRE‐POST30 comparisons revealed that SBP, DBP, and MAP were lower for EX+SAUNA. This may be indicative of a relatively greater stimulus that EX+SAUNA provided. However, PRE‐POST responses do not appear to be in support of this, as SAUNA was superior to EX+SAUNA in lowering both DBP and MAP (Table 2).

Based on the overall evidence from the present study, EX+SAUNA appears to have a relatively longer period of recovery to baseline than SAUNA. It is plausible that the combination of greater fluid loss from the relatively longer duration of heat exposure in SAUNA together with the depressive effects of prior aerobic exercise on blood pressure in EX+SAUNA contributed to this. Sauna‐induced fluid loss has been reasonably documented,^45^ and the magnitude of dehydration from the sauna has been indirectly shown to be greater than submaximal cycling exercise.^46^ This may have been what contributed to the PRE‐POST results. Notwithstanding, the effects of post‐exercise hypotension have been well established to occur even with relatively shorter bouts of exercise^47^ akin to the one used in our current study. It is thus likely that it could have led to the greater PRE‐POST30 changes in EX+SAUNA.

While the changes in some markers were still seen after 30 minutes in EX+SAUNA, the hemodynamic responses of both interventions appear to be rather comparable (Figures 2 and 3). These findings may have practical implications, especially if the amount of hemodynamic stress used in our interventions could induce adaptations. Moreover, it should be duly noted that the results seen in this current study were *in spite* of our study population being acclimated habitual sauna users. Therefore, it is plausible that a sauna naïve population may exhibit an even more pronounced cardiovascular response than what was demonstrated. The effects of regular exercise in combination with sauna use need to be investigated using long‐term trials in more representative populations, as heat therapy and the sauna may have a broader range of application than we currently recognize.

### 4.1 Limitations

In spite of the evidence provided by the current study, several limitations must be noted. From a methodological standpoint, we did not include a standalone exercise intervention. This would have enabled a comparison between EX+SAUNA and an aerobic exercise session of the same duration. However, that was not the purpose of our investigation. The scope of our data interpretation is limited due to the lack of cardiac output measurements and thermal stress (temperature) data, although we did measure heart rate and arterial stiffness.

Menstrual phase was not controlled, and we are unable to determine the degree of influence that the differences in menstrual phases may have had. However, the majority of our female participants were postmenopausal. We did not control for the water temperature of the shower, which may have had an effect on some of the results seen, although we did try to minimize this by limiting the shower time to under 30 seconds. Finally, the majority of our participants were regular sauna users (85%) of at least once a week, which limits the generalizability of our results to a broader population who do not use sauna regularly. It is thus entirely possible that the current results are specific to the population studied, though the sauna‐naïve would presumably demonstrate more pronounced responses as they would be less habituated. Nevertheless, it highlights the need for further investigation in this particular area.

## 5 CONCLUSION

The current study shows for the first time the hemodynamic differences between sauna bathing, and a combination of a short bout of aerobic exercise followed by sauna bathing, in a representative population with cardiovascular risk factors. From an acute standpoint, sauna bathing is able to elicit responses that are comparable to a combination of aerobic exercise followed by the sauna, when matched for duration. For populations who are unable to perform aerobic exercise, sauna exposure may provide some similar benefits acutely.

The long‐term adaptations of regular exercise in conjunction with passive heat such as the sauna is an area that needs more attention, and experimental trials are needed to better understand the sauna intricately, as it has shown compatibility with aerobic exercise. In the management of hypertension, emphasis is often given to improving ones' diet, performing regular exercise, or weight control. Sauna use might also be a worthwhile lifestyle treatment option to improve BP control.

## FUNDING

This research received no funding.

## CONFLICT OF INTEREST

The authors declare that there is no conflict of interest.

## AUTHOR CONTRIBUTION

Conceptualization: Earric Lee, Setor K. Kunutsor, and Jari A. Laukkanen

Data curation and integrity: Earric Lee, Tanjaniina Laukkanen, and Jari A. Laukkanen

Formal analysis: Joel Kostensalo

Investigation: Earric Lee, Tanjaniina Laukkanen, and Jari A. Laukkanen

Project administration: Tanjaniina Laukkanen, and Jari Laukkanen

Supervision: Peter Willeit, Setor K. Kunutsor and Jari A. Laukkanen

Visualization: Earric Lee, and Joel Kostensalo

Writing—original draft preparation: Earric Lee, Peter Willeit, and Jari Laukkanen

Writing—review and editing: Earric Lee, Joel Kostensalo, Peter Willeit, Setor K. Kunutsor, Francesco Zaccardi, Hassan Khan and Jari A. Laukkanen

All authors have read and approved the final version of the manuscript.

The corresponding author had full access to all of the data in this study and takes complete responsibility for the integrity of the data and the accuracy of the data analysis.

## TRANSPARENCY STATEMENT

The authors affirm that this manuscript is an honest, accurate, and transparent account of the study being reported; that no important aspects of the study have been omitted; and that any discrepancies from the study as planned have been explained.

## Data Availability

The data that support the findings of this study are available from the corresponding author upon reasonable request. The data are not publicly available due to privacy or ethical restrictions.
